# SNAP Participants’ Eating Patterns over the Benefit Month: A Time Use Perspective

**DOI:** 10.1371/journal.pone.0158422

**Published:** 2016-07-13

**Authors:** Karen S. Hamrick, Margaret Andrews

**Affiliations:** Economic Research Service, U.S. Department of Agriculture, Washington, DC, United States of America; Iowa State University, UNITED STATES

## Abstract

Individuals receiving monthly benefits through the U.S. Supplemental Nutrition Assistance Program (SNAP) often fall short of food at the end of the month and some report feelings of hunger. To investigate this situation, we used time diaries from the 2006–08 American Time Use Survey and Eating & Health Module to identify the timing of days where respondents reported no eating occurrences. Analysis includes descriptive statistics, a logit model, and a simulated benefit month. We found that SNAP participants were increasingly more likely than nonparticipants to report a day with no eating occurrences over the benefit issuance cycle. This supports the view that there is a monthly cycle in food consumption associated with the SNAP monthly benefit issuance policy.

## Introduction

Given the national concern regarding the health effects of obesity, it is difficult for many to view hunger as a serious problem in the United States. In addition, social welfare policy is heavily oriented toward food assistance programs. Predominant among these programs is the Supplemental Nutrition Assistance Program, SNAP, formerly known as the Food Stamp Program, which provides low-income households with monthly benefits to increase their food purchasing power and prevent hunger. Benefits can only be spent on food purchases. In FY2015, $74 billion in SNAP benefits were distributed by the U.S. Government to an average monthly participant base of 45.8 million people.

It has been a longstanding observation that those receiving SNAP benefits tend to have serious problems procuring food at the end of the month. Benefits are spent rapidly with an average of 59 percent spent within the first week of issuance, with a quarter of all households exhausting benefits within the week [1]. Even though benefits are meant to be supplemental, participants often face unexpected budgetary demands or may have miscalculated their meal planning, and thus lack resources to procure food when benefits run out. Alternative resources, such as food pantries and soup kitchens, often report added demand at the end of the month.

Using time diaries from the 2006–08 American Time Use Survey (ATUS) and Eating & Health Module (EHM) data, we analyzed eating occurrences over the SNAP benefit cycle. To proxy hunger, we identified days where there were no eating occurrences. We looked at both primary eating and drinking, that is, eating and drinking as a main activity, and secondary eating, eating while doing something else. Respondents identified with no eating occurrences reported zero primary eating and drinking and zero secondary eating over the entire day. By estimating the ATUS respondent’s issuance date, we were able to approximate the ATUS diary date in relation to the monthly benefit cycle. We analyzed descriptive statistics, estimated a logit model to control for various factors, and then simulated the benefit month in order to compare how SNAP recipients differ from low-income non-SNAP individuals and higher-income individuals in the experience of not eating for a whole day.

This research is the first to look at the SNAP benefit cycle using time diaries. The ATUS data has the benefit of being nationally representative of individual eating behavior as opposed to the household expenditure or grocery scanner data used in some prior studies. Time diaries also have the benefit of being a neutral method of collecting information on individual eating behaviors, reducing social bias in responses. In using the time diary data, our work acknowledges the extent to which the SNAP benefit cycle does not strictly coincide with the calendar month and we implement an approximation to account for difference in state SNAP benefit issuance practices.

### Background

SNAP is the largest federal food assistance program. It was originally authorized as the Food Stamp Act of 1964, and has been reauthorized with various legislation including the U.S. Farm Bills. On October 1, 2008, the program name changed from the Food Stamp Program (FSP) to the Supplemental Nutrition Assistance Program. Although the program name was FSP during most of the analysis period, the program will be referred to here by the current name, SNAP.

By 2002, all states operated Electronic Benefit Transfer (EBT) systems where benefits are issued automatically to accounts accessed by participants at qualified food retailers using an EBT card similar to a debit card. Paper food stamp coupons are no longer in use. For the program participant, EBT has the advantage of making benefits immediately available instead of waiting for the coupons to be picked up or arrive by mail. EBT also reduces the risk of loss or theft as well as reducing stigma. SNAP is a Federal program, but administered by the states. Benefits are issued monthly according to different dates chosen by the states. (For more information on SNAP, see http://www.fns.usda.gov/snap/supplemental-nutrition-assistance-program-snap and http://www.ers.usda.gov/topics/food-nutrition-assistance/supplemental-nutrition-assistance-program-(snap).aspx)

### Benefit Cycle

The end-of-the-month problem, that food runs out at the end of the month after a paycheck or assistance benefits, has been well documented though few studies have explicitly examined the heterogeneity of the SNAP benefit issuance cycle. One of the first studies to systematically look at the issue is Thompson et al. [2]. They looked at public assistance issuance date and soup kitchen usage in New York state. They found that soup kitchen use increased over the month as measured by the mean number of meals served.

Wilde and Ranney [3] studied the monthly cycle in food expenditure and food intake using the Consumer Expenditure Survey (1988–92 data) and the Continuing Survey of Food Intake by Individuals (CSFII, 1989–91 data). First they categorized households as being frequent grocery shoppers, that is, they grocery shopped more than once a month, and infrequent shoppers, who grocery shopped at most once a month. They found that 42 percent of food stamp households were infrequent shoppers, and that infrequent shoppers were more likely to experience a monthly food cycle, that is, a drop in food energy intake at the end of the benefit month. They also found that there is a sharp spike in food expenditures in the first three days after benefit issuance.

Shapiro [4] used the CSFII dietary recall data together with survey information on the date of food stamp receipt to estimate a decline in calorie intake of food stamp participants of between 0.32 to 0.4 percent per day after benefits were received. He also used data from the Evaluation of the EBT Expansion in Maryland survey to examine how food stamp participants are willing to trade off uncertain consumption in the future versus more certain consumption today. In doing so he found that food stamp participants have a high discount rate—meaning that they are impatient in the short-run favoring current over future consumption. He argued that this explains to some extent the decline in food intake over the benefit month measured both as food value and caloric intake.

Tarasuk et al. [5] studied dietary intake among Canadian food-insecure women with children over the month after the household received their primary source of income. They found that over the month, “women with moderate or severe food insecurity exhibited declines in energy, carbohydrate, and vitamin B-6, and fruit and vegetable intakes” (p. 1984).

Weinstein et al. [6] surveyed households in Hartford, Connecticut to investigate whether food insecurity varies within a month. They found that households visited during the latter third of the month were 5.5 times more likely to be food insecure than those visited earlier in the month, and food stamp participating households were even more likely to report food insecurity during the last third of the month.

Calloway et al. [7] recruited parents in a city in the midwestern United States to participate in a survey on hunger-coping behaviors and other food-related issues, and examined the relationship between SNAP benefit duration—the number of weeks the benefit lasted—and various outcome variables. They found that the longer the benefits duration, the less likely the family experienced low food security or physiological hunger symptoms. They attributed longer benefit duration to allotment adequacy or more efficient use of benefits. In addition, families who also participated in other assistance programs such as WIC (Special Supplemental Nutrition Program for Women, Infants, and Children) tended to have longer SNAP benefit durations.

Todd [8] used 2007–10 National Health and Nutrition Examination Survey (NHANES) data to examine food intake over the benefit month before and after the American Recovery and Reinvestment Act of 2009 that provided for a boost in SNAP benefits starting April 2009 through October 2013. She found that evidence of a benefit cycle before the Act took effect, but found that after implementation the increased benefits appeared to smooth food intake over the benefit month.

### Benefit Cycle and Health

A particular health concern in terms of the benefit cycle is whether or not the “feast or famine” cycle with “binge” or compensatory eating directly after benefit receipt and reduced intake at the end of the month is associated with or contributes to obesity. Several studies have investigated this issue. Chen et al. [9] using 1994–96 CSFII data concluded that participating in the Food Stamp Program is related to obesity in women but not men. Zagorsky and Smith [10], using data from the 1989–2002 National Longitudinal Survey of Youth, found that body mass index (BMI) increased before, during, and after participants are in the program, with the largest increase occurring during program participation. They conclude that “…while the Food Stamp Program, as currently structured, reduces one problem, food insecurity, it may inadvertently exacerbate another problem, obesity” (p. 257). However, Almada et al. [11] found that when misreporting of program participation is taken into account, there was “…no evidence that SNAP participation significantly increases the probability of being obese or overweight (either overall or among men and women separately)” (page 4).

Seligman et al. [12] analyzed whether there is a relationship between the “pay cycle” and hospital admissions for hypoglycemia using data from California’s Office of Statewide Health Planning and Development. “For people with diabetes, a stable dose of medication for glycemic control coupled with a temporary reduction in food intake would be expected to result in increased risk for hypoglycemia (p. 177).” They found that admissions for hypoglycemia were more common among low-income individuals, and admissions increased from the first to the last week of the month, whereas there was no increase over the month of the control condition, appendicitis.

### Benefit Cycle and Retail Stores

Over 2010–11, there was national media attention to the phenomenon of SNAP recipients flooding stores at midnight on their benefit issuance day [13–17]. At midnight, participants’ electronic benefit transfer (EBT) accounts are automatically credited with the new month’s benefits. 24-hour stores such as Wal-Mart, Kroger, Kmart, Target, and Costco plan for the rush by having additional staff and inventory on hand.

Researchers Hastings and Washington [18] used grocery store scanner data to analyze participants’ purchases over the benefit cycle in Nevada. Nevada issued all food stamp benefits on the first day of the month, so all participants were on the same benefit cycle. Among their findings were that food prices move pro-cyclically with demand, that is, that prices are higher at benefit issuance. They also found that the decrease in food expenditures over the month is due to reduced food quantities and not reduced food quality. They found “…no evidence that the food spending cycle is driven by a desire for variation in food products consumed throughout the month” (p. 144).

Damon et al. [19] also used scanner data to examine food expenditures in SNAP households. They found that SNAP households had a decline in food expenditures over the month, and shopped more at grocery stores and mass/club/superstores early in the month, and then at smaller convenience stores and food-away-from-home establishments later in the benefit month. Castellari et al. [20] also used scanner data to examine if what day of the week the benefits were received made a difference in purchases. They found that “monthly purchases of beer are higher within the same households when the benefits are more likely to have been distributed on weekends” (no page number). They suggest that distributing benefits more than once a month may improve participants’ welfare.

### Policy Issues

The 2008 Farm Bill (“Food, Conservation, and Energy Act of 2008,” http://www.gpo.gov/fdsys/pkg/PLAW-110publ246/pdf/PLAW-110publ246.pdf) amended the previous Farm Bill, passed in 2002, by stipulating that SNAP benefits could not be issued more than once a month: “(B) MULTIPLE ISSUANCES. The procedure may include issuing benefits to a household in more than 1 issuance during a month only when a benefit correction is necessary.” (Section 4113. Clarification of Split Issuance) The 2014 Farm Bill (http://www.gpo.gov/fdsys/pkg/BILLS-113hr2642enr/pdf/BILLS-113hr2642enr.pdf) did not amend this provision of SNAP requirements.

Before the 2008 Farm Bill, there was discussion among program practitioners, researchers, and policy analysts that food stamp participants should be able to opt for semi-monthly benefit issuance in order to avoid the end-of-the-month problem [21]. With EBT cards, this benefit election would be automatic and would pose minimal burden on states’ administrative offices. The monthly benefit amount would be the same, but divided into two payments. Semi-monthly benefits would also help retailers in spreading out sales over the month. However, the 2008 Farm Bill removed the possibility of semi-monthly benefit issuance. Discussion has continued on the need for this option [22,23]. In response to retailers’ request for spreading out benefit issuance, some states have increased the number of days in their issuance schedules. So, although participants receive benefits only once a month, different participants receive benefits on different days of the month, avoiding a rush on retail stores on the first of the month.

### Data

Unlike previous research that used food diary, consumer expenditure, or scanner data, we used time use data to study the benefit cycle. The Bureau of Labor Statistics’ American Time Use Survey (stats.bls.gov/tus/) is a continuous survey that began in 2003. Interviews are conducted by the U.S. Census Bureau, and are done nearly every day of the year. One individual age 15 or older from each sampled household is interviewed about his or her activities for the 24-hour period from 4 am the day before the interview to 4 am of the interview day. Survey respondents are asked to identify their primary activity (if they were engaged in more than one activity at a time), where they were, and whom they were with for most diary activities. The ATUS also includes demographic, labor force participation, and household information, along with a limited amount of geographical information. The ATUS Respondent, Roster, Activity, Activity Summary, ATUS-Current Population Survey, and Methodology Case files were used for our research.

The Eating & Health Module (EHM, ers.usda.gov/data-products/eating-and-health-module-(atus)) was a supplement to the ATUS over 2006–08. The EHM included questions on secondary eating (that is, eating while doing something the respondent considered a primary activity), secondary drinking beverages, Supplemental Nutrition Assistance Program/Food Stamp Program participation, income, general health, and height and weight. Specifically, the EHM asked, *In the past 30 days*, *did you or anyone in your household get food stamp benefits*? During most of the fielding of the EHM the program was known as “food stamps” and the name change to SNAP was effective October 1, 2008, although soon after it was still generally referred to as “food stamps.” Over 2006–08, the ATUS and EHM resulted in 37,832 completed interviews of individuals age 15 or over. Extensive descriptive estimates of time use patterns of SNAP participants and others is in Hamrick et al. [24].

We excluded respondents with bad diaries, resulting in 37,554 completed interviews. Bad diaries are those flagged by the interviewers as: respondent intentionally providing wrong answer; respondent trying to provide correct answer, but could not correctly remember his/her activities; respondent deliberately reporting very long duration activities; or other reason. The EHM Respondent and Replicate weights files were used.

The 2006–08 EHM data are the most recent time use data available to do benefit cycle analysis using the ATUS. We did not restrict the sample to those in households eligible for SNAP in order to avoid small sample problems with large standard errors. Also, SNAP eligibility is difficult to determine as categorical eligibility rules allow states to elect from a wide range of the income thresholds (130–200 percent of the federal poverty level) for SNAP benefit eligibility resulting in some states having a higher income threshold than in other states [25].

Estimation procedures outlined in the *ATUS User’s Guide* [26] and the *Eating & Health Module User’s Guide* [27] were followed. All estimates presented were weighted to be nationally representative estimates. Averages were calculated as the mean. Standard errors were calculated according to Section 7.5 of the *ATUS User’s Guide*, using the balanced repeated replication method and the EHM Replicate Weights file. A 90-percent level of confidence was used to determine whether estimates were statistically different, both by analysis of the confidence intervals as well as by t-test. All differences between estimates discussed in the text are statistically different at the 90 percent level unless stated as not statistically different. The 90-percent level is the standard level of confidence used with the Current Population Survey (CPS) and ATUS household surveys [28]. Estimates were done in SAS 9.2. We did not use the more recent SAS 9.4 although it was available to us, as we discovered a glitch in PROC SURVEYLOGISTIC, which we reported to SAS Institute Inc.

The data were pooled over 2006–08, and so estimates are for an average day over 2006–08. Unweighted data would produce averages for ATUS respondents on their diary day, and weighted estimates are averages for the U.S. population age 15 and over on an average day over 2006–08.

We used the ATUS time diaries to determine whether an individual’s time in primary eating or drinking, that is, eating/drinking as a main activity, plus time in secondary eating, eating while doing something else, was zero minutes. We defined primary eating and drinking as total time spent in activities 050202 (Eating and drinking as part of job, such as a business lunch), 110101 (Eating and drinking), 110199 (Eating and drinking not elsewhere classified), and 119999 (also Eating and drinking not elsewhere classified). Eating and/or drinking is self-reported by the respondent, and includes the activities of eating only, drinking only (which includes all beverages—coffee, tea, juice, soft drinks, alcohol), or eating and drinking together. Zero time spent in eating/drinking—no primary eating/drinking and no secondary eating—was considered no eating activity for the day.

On any given day, about one percent of the U.S. population age 15 and over does not engage in primary eating/drinking activity or in secondary eating. There are a variety of reasons individuals may refrain from eating, including medical (illness or preparation for medical procedure), restriction of calories, being busy or stressed, or lack of resources to purchase food. To report no eating may be an indicator of household food need, especially in low-income households. The Current Population Survey Food Security Supplement asks respondents if adults or children did not “eat for a whole day because there was not enough money for food,” and responses to this question are used as an indicator of “very low food security.” The underlying Food Security Scale classifies this situation as an indicator of the most severe condition of food security [29,30].

The EHM variable on household SNAP participation was used to determine SNAP participants. Low-income non-SNAP individuals were identified using the EHM variables on income level. The EHM asked respondents if their household income in the past 30 days was above/below a dollar amount that was equal to 185 percent of the poverty threshold for their household composition. If they answered “below,” they were asked if household income fell below the 130 percent threshold. Both of these thresholds are used by USDA food assistance programs to determine income eligibility. We used the more-inclusive 185 percent measure to identify individuals who might qualify for SNAP. However, we do not define this low-income group as SNAP-eligible given complications of categorical eligibility rules, discussed above, and other eligibility factors not associated with income. In addition to the EHM income variables, we use household income data from the CPS. Because all ATUS respondents were previously in a CPS panel, those data are available for use in our logistic regression.

The advantage of using the ATUS and EHM data is that time diaries are considered a neutral method of collecting data on time spent in various activities. They are less subject to under- and overreporting including social desirability bias than surveys that ask for frequency of an activity or for estimates of time spent on specific activities [31,32]. In addition, the ATUS interviews respondents nearly every day of the year. Consequently, the ATUS provide the daily data needed to analyze days since benefits were received.

### SNAP Issuance Date

SNAP benefits are issued by states and the District of Columbia using a debit card system known as Electronic Benefit Transfer (EBT) cards. Only a small number of states issue benefits to all participants on the same day each month (usually the first of the month). The other states have a variety of issuance schedules where the specific day of the month participants receive benefits may depend on the first letter of their last name, the first or last digit of their Social Security number, or their case number.

With the ATUS and EHM data, we know the day of the respondent’s time diary, and we know whether or not their household received SNAP benefits in the month before the ATUS interview. However, if the respondent lived in a state with more than one issuance day, we do not know exactly what day benefits were received. Consequently, we needed to impute an issuance day for respondents in these states. We used the mid-point of the range of issuance days as the imputed issuance day. See S1 Appendix for a listing of states’ issuance schedules and our imputed issuance days. We defined week 1 as 1–7 days since benefit issuance; week 2 as 8–14 days since issuance; week 3 as 15–21 days since issuance; and week 4 as the remainder of days until the next issuance day. The number of days in week 4 varies by month. For states with a longer window of issuance, our procedure risks a possible misclassification of issuance week.

By imputing an issuance day and knowing the diary day, we estimated how many days have passed since benefit issuance. This way we “lined up” the respondents as to the benefit issuance days and not calendar days. We then analyzed the time use patterns, and in particular, eating occurrences in relation to issuance days.

We assigned an issuance day to non-SNAP individuals to test the hypothesis that SNAP participants react differently to the time elapsed since issuance than others. Although our estimate for days since issuance is hypothetical for non-SNAP participants we include it as a variable in order to eliminate the possibility that there is an unidentified factor associated with the SNAP issuance cycle that affects the behavior of both SNAP and non-SNAP groups.

### Descriptive Statistics

The percent of SNAP participants who did not report any primary eating/drinking occurrences or any secondary eating occurrences is low in the first week after benefit issuance, less than one percent (0.65 percent, Table 1). However, over the course of the benefit month, the percent with no reported eating rises to 1.68 percent in week 4. However, due to large standard errors and the resulting large confidence intervals, there is no statistical difference between any of the two adjacent weeks.

**Table 1:** Percent of group that reported no primary eating/drinking or secondary eating on an average day, 2006–08, age 15 and over, by week since SNAP issuance.

	Week 1	Week 2	Week 3	Week 4*
**SNAP participants**	**0.65**	**1.48**	**0.83**	**1.68**
90% confidence interval	[0.26,1.05]	[0.41,2.55]	[0.12,1.54]	[0.78,2.59]
N	741	579	547	744
**Low-income non-SNAP**	**1.13**	**0.47**	**1.14**	**0.58**
90% confidence interval	[0.56,1.69]	[0.18,0.76]	[0.60,1.67]	[0.33,0.84]
N	2,483	1,943	1,822	2,388
**High income (> 185% poverty threshold) non-SNAP**	**0.39**	**0.36**	**0.65**	**0.46**
90% confidence interval	[0.29,0.49]	[0.21,0.50]	[0.34,0.96]	[0.29,0.62]
N	6,892	5,664	5,067	6,874
**Total population**	**0.73**	**0.51**	**0.94**	**0.67**
90% confidence interval	[0.55,0.91]	[0.36,0.67]	[0.67,1.21]	[0.52,0.82]
N	10,662	8,566	7,826	10,500

90% confidence interval in brackets [lower bound, upper bound]. Source: Authors’ estimates using 2006–08 American Time Use Survey and Eating & Health Module data.

* Week 4 includes calendar month days 29, 30, and 31 for those months that contain those days.

We also estimated hypothetical SNAP issuance days for nonparticipants in order to compare their rate of reporting eating occurrences over the benefit cycle with the SNAP individuals’ patterns. Low-income individuals, those with household income less than 185 percent of the poverty threshold, who were not participating in SNAP had 1.13 percent of the group in week 1 report no eating occurrences. Although the percent of those with no eating occurrences appears to move over the weeks, the estimates are not statistically different as their confidence intervals overlap and t-tests produced the same result. Consequently, the rate of those with no eating occurrences is about the same over the month, as expected since the SNAP benefit issuance day does not apply to them.

High-income (household income greater than 185 percent of the poverty threshold) individuals not participating in SNAP had a low rate of those not reporting eating occurrences in week 1, 0.39 percent. The rate is about the same (not statistically different) over the weeks.

**Fig 1:**
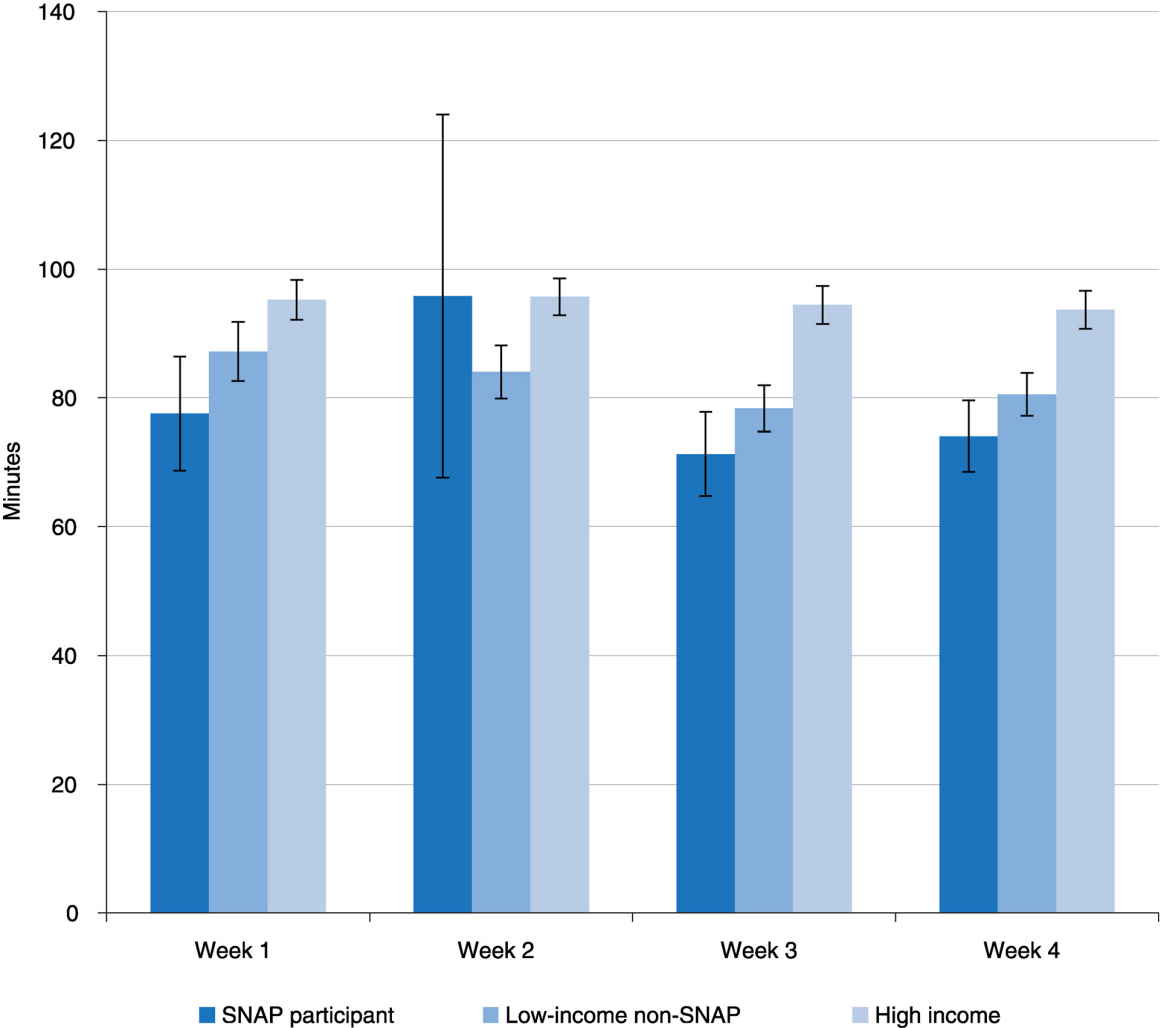
Time spent in eating (primary eating/drinking and secondary eating), in minutes, on an average day 2006–08, age 15 and over. Week 4 includes calendar month days 29, 30, and 31 for those months that contain those days. Black lines are 90% confidence intervals for each estimate. Source: Authors’ estimates using 2006–08 American Time Use Survey and Eating & Health Module data.

Looking at the average times spent in primary eating/drinking and secondary eating (Fig 1), we see that SNAP participants had a relatively short duration average time spent in eating in week 1, a relatively long duration in week 2, then short durations in weeks 3 and 4. However, again due to large standard errors we cannot make a strong statement about eating time duration over the benefit cycle for either SNAP participants or for the low-income non-SNAP group using descriptive statistics. The estimated total time spent eating for SNAP participants in week 2 has a particularly large standard error. This is because this group has disproportionately more respondents who reported very long eating times, that is, more than 600 minutes, which created a dispersed distribution. Such long eating times are typically reported when a respondent attends an event such as a reception, or reports engaging in secondary eating “all day.” This resulted in a large standard error relative to the estimates for the other weeks.

Because of large standard errors in the descriptive statistics, another method of analysis is needed to better determine the story told by the time use data as to eating patterns over the benefit cycle.

### Logistic Regression Model

We estimated a model of the likelihood of an individual going an entire day without eating. The model developed is the probability of

Pr(y = 1|x) = F(xβ)

where:

*y*_*i*_ = 1 no primary eating/drinking or secondary eating occurrences

*y*_*i*_ = 0 at least one primary eating/drinking occurrence and/or secondary eating occurrence

*F* = logistic cumulative distribution function

**x =** matrix of individual-level variables

$$p_{i}\;=\text{ Pr}\left(y_{i}=1|x_{i}\right)\text{ if}\;y_{i}\;=\;1$$

$$p_{i}=1\;–\text{ Pr}\left(y_{i}=1|x_{i}\right)\text{ if }y_{i}\;=\;0$$

and where the matrix **x** contains variables pertaining to SNAP, calendar characteristics, household characteristics, individual characteristics, and geographical information. Individual subscripts omitted for presentation clarity. This results in the linear logistic model:

$$\text{logit}\left(p\right)\;=\text{ log}\left[\frac{p}{\left(1-p\right)}\right]\;=\;\alpha \;+\;\beta \prime x$$

#### Eating occurrences

We defined no eating as no (zero minutes) primary eating or drinking—the ATUS does not distinguish between primary eating and primary drinking beverages—and no (zero minutes) of secondary eating. Although 4 percent of Americans age 15 and over had no primary eating/drinking occurrences on an average day over 2006–08, less than one percent (0.71 percent) had no eating under our definition that includes secondary eating, making this a rare situation.

Because not eating for a whole day is a rare situation, we risk bias in using the standard logistic regression model [33]. However, estimation using a rare event approach (such as the Firth method) poses a problem with our data, at least with existing software. The ATUS has a complex sampling design, both stratified and clustered, and so is nonrandom. The ATUS treatment for this situation is the use of balanced replicate weights (BRR). The BRR method uses variation between primary sampling units within strata to estimate standard errors. Without BRR, the standard errors are underestimated. Available estimation methods for the Firth method do not accommodate the probability weights needed for estimation using the BRR, and so will produce underestimates of the standard errors. As a result, we use the standard logistical regression model, estimated with BRR in order to obtain correct estimates of standard errors. We also performed a “rare events” estimation of our model using the Firth method as a robustness test, which is discussed below.

#### SNAP characteristics

The model included an indicator of SNAP participation, so the reference group is SNAP non-participants. Also included was the log of the number of days since benefit issuance, and also an interaction term between SNAP participation and the log variable. The log of the number of days since benefit issuance was used to capture the steep drawdown pattern of SNAP benefits redemption—in FY2009, 21 percent of benefits are redeemed on the first day of issuance, 59 percent at the end of the first week, and 79 percent at the end of the first two weeks [1]. The interaction term captures whether or not the effect of days since issuance is different for SNAP participants than others, or more generally, whether the marginal effect is different for SNAP participants than others at different values of issuance dates [34].

#### Calendar variables

We pooled the 2006–08 ATUS and EHM data, and because of this, we included dummy variables for 2006 and for 2007, with year 2008 as the reference group. These year dummies will control for any year-to-year effects, and in particular, the recent recession (December 2007 to June 2009, see National Bureau of Economic Research, U.S. Business Cycle Expansions and Contractions, http://www.nber.org/cycles/cyclesmain.html). In addition, we added day-of-the week dummies for Saturday, Sunday, and holidays (New Year’s Day, Easter, Memorial Day, Independence Day/Fourth of July, Labor Day, Thanksgiving Day, and Christmas Day) as eating patterns may be different on these days. We included season dummies for spring (March, April, May), summer (June, July, August), and fall (September, October, November), with winter (December, January, February) as the reference group.

#### Household characteristics

In addition to household income, we included the number of adults in the household and the number of children in the household. Household income clearly is an important determinant of food sufficiency, but so also is household composition. Previous research found that adults in a food insufficient household will go without in order for their children to have meals [35]. Also included is whether or not the individual’s spouse/partner is present in the household, with the reference group as no spouse/partner in the household. Finally, an indicator of whether or not the individual owns the home is included.

The Current Population Survey (CPS) variable HUFAMINC was used in the logit estimation in order to analyze a range of income levels. (All ATUS respondents were previously in the Current Population Survey and sampling for the ATUS was done after respondents’ final outrotation from the CPS. The ATUS interview is usually 2–5 months after the final CPS interview.) In the descriptive statistics and simulated results sections, the ATUS Eating & Health Module variable EEINCOME1 was used to identify individuals in households with incomes greater than 185 percent of the poverty threshold or less than 185 percent of the poverty threshold. The advantage of HUFAMINC is that it has 16 income categories, but it is collected in the first month of the CPS, making it more than 16 months old when the respondent is surveyed for the ATUS. The advantage of the EEINCOME1 variable is that it is current with the time diary, although it does not have the detail of HUFAMINC. We use both income measures in our analysis to take advantage of each measure’s strength.

#### Individual characteristics

Included are a standard group of demographic and labor force characteristics: gender (indicator for female); age (age in years, indicator for teens age 15–19, and indicator for seniors age 65 or over); indicator for disabled; education level (high school graduate, some college, college degree or advanced degree); race/ethnicity (African American, Asian, Hispanic); and employment status (employed, retired). The reference group is then male, age 20–64 years old, not disabled, has less than a high school diploma, is white, non-Hispanic, and is not employed and not retired.

#### Region

As there may be regional effects, we included indicators for metropolitan/nonmetropolitan residence (metro, with nonmetro as the reference group) and for Census region (West, South, and Northeast, with Midwest as the reference group).

The resulting model is a logistic regression on the likelihood of no primary eating/drinking and no secondary eating, as explained by SNAP participation, days since issuance and an interaction term, along with controls for day of week, season, year, and household, personal, and geographic factors.

Using the estimated model and the estimated means of the model variables for each group (see S2 Appendix), we simulated a full benefit cycle month of daily probability of not reporting any eating occurrences.

## Results

Results of the estimated logit model of the probability of not eating over the day are in Table 2. Regardless of the point in the benefit cycle, being a SNAP participant lowers the likelihood of no eating over the day (coefficient is -1.1027 and significant, odds ratio is 0.332), and the log of the days since benefit issuance, regardless of SNAP participation status, appears to lower the likelihood (-0.1435 coefficient, 0.866 odds ratio), although the coefficient is not significant so it may not have any effect. So far this does not support our hypothesis that SNAP individuals have a greater likelihood of not eating towards the end of the benefit month. However, these effects are in conjunction with the interaction variable, log of the days since issuance multiplied by SNAP participation (log of days since issuance for SNAP participants, zero for others), which is positive, fairly large, and significant (0.4444). For a given number of days since issuance, a SNAP participant would have odds 1.560 higher than the reference group. We need to determine the combined effect of these variables, however, a simple addition of the odds ratios is not recommended [36]. We will address the net effect of the SNAP participation effect, but after discussing the impact of other variables.

**Table 2:** Logit model of the probability of not eating over an average day, 2006–08.

	Maximum Likelihood Estimate	Standard Error	Wald Chi Squared	Probability Chi Sq	Odds Ratio Estimate	90% Wald CI min	90% Wald CI max
Intercept	-3.5906	0.6702	28.7057	<.0001			
**SNAP characteristics**							
SNAP/FSP participant	-1.1027	0.5899	3.4938	0.0616	0.332	0.126	0.876
ln(days since issuance)	-0.1435	0.1071	1.7961	0.1802	0.866	0.726	1.033
ln(days since issuance) times SNAP/FSP participant	0.4444	0.2225	3.9910	0.0457	1.560	1.082	2.249
**Calendar variables**							
Year 2006	-0.0185	0.2339	0.0062	0.9370	0.982	0.668	1.442
Year 2007	-0.1747	0.2049	0.7272	0.3938	0.840	0.599	1.176
Saturday	0.4930	0.1964	6.2986	0.0121	1.637	1.185	2.262
Sunday	0.2297	0.2153	1.1386	0.2859	1.258	0.883	1.793
Holiday	0.6066	1.1522	0.2771	0.5986	1.834	0.276	12.204
Spring	-0.3262	0.2152	2.2981	0.1295	0.722	0.507	1.028
Summer	0.0081	0.2175	0.0014	0.9702	1.008	0.705	1.442
Fall	0.0860	0.2559	0.1130	0.7368	1.090	0.715	1.660
**Household characteristics**							
Family income category (1–16)	-0.0535	0.0263	4.1417	0.0418	0.948	0.908	0.990
Number of adults in household	-0.1038	0.0874	1.4120	0.2347	0.901	0.781	1.041
Number of children in household	-0.0110	0.0810	0.0185	0.8919	0.989	0.866	1.130
Spouse/partner in household	-0.3717	0.2178	2.9135	0.0878	0.690	0.482	0.987
Own home	-0.0237	0.2075	0.0131	0.9089	0.977	0.694	1.374
**Individual characteristics**							
Female	-0.0951	0.1734	0.3009	0.5833	0.909	0.684	1.209
Employed	-0.0184	0.2462	0.0056	0.9404	0.982	0.655	1.472
Age	0.0042	0.0093	0.2046	0.6511	1.004	0.989	1.020
Teen (age 15–19 years)	-0.0347	0.4552	0.0058	0.9392	0.966	0.457	2.042
Age 65 years or over	-0.3583	0.3757	0.9095	0.3403	0.699	0.377	1.296
Retired	-0.4637	0.4291	1.1681	0.2798	0.629	0.311	1.274
Disabled	-0.3039	0.3996	0.5786	0.4469	0.738	0.382	1.424
High school diploma	0.0618	0.2607	0.0562	0.8126	1.064	0.693	1.633
Some college	-0.4021	0.2657	2.2914	0.1301	0.669	0.432	1.035
College or advanced degree	-0.8644	0.3423	6.3750	0.0116	0.421	0.240	0.740
African American	0.8614	0.2250	14.6540	0.0001	2.367	1.634	3.427
Asian	0.4614	0.6900	0.4472	0.5037	1.586	0.510	4.935
Hispanic	0.2041	0.3072	0.4416	0.5063	1.226	0.740	2.033
**Region**							
Metropolitan residence	-0.0004	0.2136	0.0000	0.9986	1.000	0.703	1.420
West	-0.2240	0.3028	0.5473	0.4594	0.799	0.486	1.315
South	-0.2218	0.2186	1.0300	0.3102	0.801	0.559	1.148
Northeast	-0.1803	0.2420	0.5551	0.4562	0.835	0.561	1.243
N	32,060						
Percent of observations that have no eating occurrences	0.7%						
Likelihood Ratio, Pr>ChiSq	<.0001						
Score, Pr>ChiSq	<.0001						
Wald, Pr>ChiSq	<.0001						
Association of predicted and observed: 62.6 percent Concordant, 26.5 Discordant, 10.9 Tied.							

Note: Age 15 and over. 90% Wald CI min = the minimum value of the Wald confidence interval at the 90% level. 90% Wald CI max = the maximum value of the Wald confidence interval at the 90% level. Family income categories are: 1 = Less than $5,000; 2 = $5,000 to $7,499; 3 = $7,500 to $9,999; 4 = $10,000 to $12,499; 5 = $12,500 to $14,999; 6 = $15,000 to $19,999; 7 = $20,000 to $24,999; 8 = $25,000 to $29,999; 9 = $30,000 to $34,999; 10 = $35,000 to $39,999; 11 = $40,000 to $49,999; 12 = $50,000 to $59,999; 13 = $60,000 to $74,999; 14 = $75,000 to $99,999; 15 = 100,000 to $149,999; and 16 = $150,000 and over. Reference group is SNAP/FSP non-participant, year 2008, non-holiday weekday, winter, no spouse/partner in home, do not own home, male, not employed, age 20–64 years, not retired, not disabled, less than high school diploma, white non-Hispanic (non-African American, non-Asian, non-Hispanic), non-metropolitan area, and Midwest. Concordant-Discordant is a measure of the model’s performance. For more information, see Paul D. Allison, Logistic Regression Using the SAS System: Theory and Application, Cary, NC: SAS Institute Inc., 1999. Source: Authors’ estimates using 2006–08 American Time Use Survey and Eating & Health Module data.

Of the calendar variables, Saturday had the largest contribution to the likelihood of not eating over the day for everyone, not just the SNAP participants. If the average day is a Saturday, the likelihood of no eating is increased, and the odds are 1.637 higher than the reference group. Perhaps since Saturday is a less scheduled day for many, eating may be second to sleeping or running errands. For those who are low-income, Saturday may not provide the resources that the weekday does, and adults in food insecure homes may forfeit their meals so their children may eat, as the children would not be getting school meals on a Saturday.

As expected, the higher the family income, the less likely the individual goes the day without eating. Each move up the income categories decreases the probability of no eating over the day. Also as expected, the higher the education level, the lower the probability of no eating over the day. Those with a college or advanced degree had odds of only 0.421 to 1. Being African American greatly increased the probability of not eating over the day, with a significant coefficient of 0.8614 and an odds ratio of 2.367.

Although few of the model’s variables were significant at the 90 percent level, the model overall did well in terms of fit and performance, with a concordance level of 62.6 percent.

### Simulated Benefit Month

**Fig 2:**
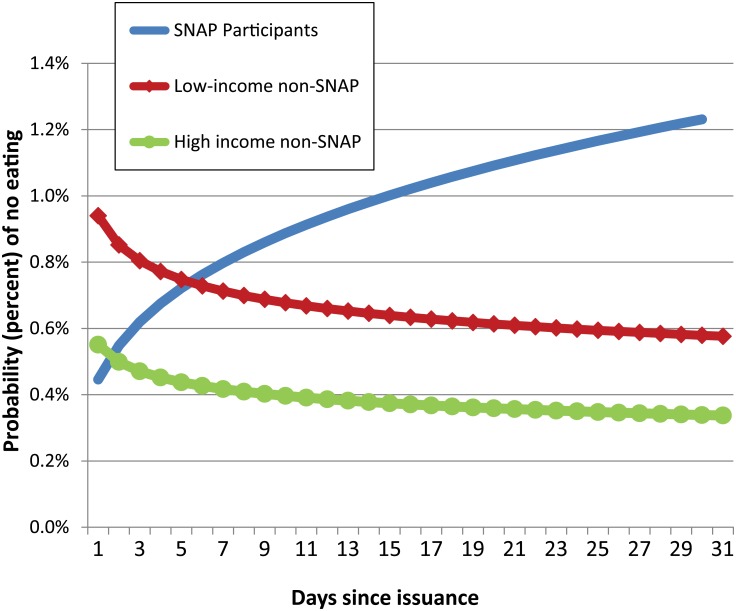
Probability of not reporting any eating occasions, by days since SNAP issuance. Source: Authors’ estimates using 2006–08 American Time Use Survey and Eating & Health Module data.

In order to understand the net effect of the factors affecting the probability of not eating on the average day, and also to understand the net effect of the SNAP and days since issuance variables, we simulated a benefit month. We used the estimated model in table 2 and the sub-sample averages (S2 Appendix) to calculate the probabilities of not eating by day since issuance for (1) those who were on SNAP, (2) those who were low-income (less than 185 percent of the poverty threshold) but not participating in SNAP, and (3) those who were high income (more than 185 percent of the poverty threshold) and not participating in SNAP. We calculated these probabilities for days 1–31 in the simulated benefit month. These probabilities are presented in Fig 2. The graph represents the predictive values based on each group’s averages. We used the one model above, and not a separate model for each subgroup. As estimated, our model includes a large array of household and individual controls that allow us to exploit the covariate differences among the three groups in the simulation.

SNAP participants have an increasing probability of having a day with no eating occurrences over the benefit issuance cycle. Looking at the probabilities charted over the month shows the net effect of the apparent contradiction of the estimated logit coefficients on the SNAP and days since issuance variables. Whereas the other groups, not affected by benefit issuance day, have essentially level probabilities over the month, while the SNAP participants have a greatly changed and increased probability from the beginning to the end of the month. Directly after benefit issuance, SNAP participants spend their benefits creating an abundance of food in the household. By the second week, much of the food is consumed, increasing the likelihood of not eating on a given day.

## Limitations

The main limitation in our research is the imputation of SNAP benefit issuance days for some states. The effect of the imputation on our estimate of days since issuance is more serious in states with longer issuance spans. We did a robustness test of estimating the model using only states with actual issuance days in the first two weeks of the month, which is most states (41 of the 50 states plus the District of Columbia, see S3 Appendix). The resulting coefficients were very similar to our estimates above using the full sample, indicating that any errors in imputation of issuance days is likely not affecting our overall results.

In addition, we estimated the model using the Firth method to correct for “rare events” (see S4 Appendix). This was done in SAS 9.2 using PROC LOGISTIC with the Firth model option. The coefficients are identical to our estimates in Table 2 and so do not indicate serious bias. Our use of the Firth method did not correct for complex sample design, and thus the standard errors are seriously biased downward, yielding almost all of the coefficients as significant at the 99-percent level. Because we know that these standard errors are underestimates and because the coefficient estimates are similar using both approaches, this robustness test favors our use of the standard logistic model with balanced replicate weights.

Underreporting of SNAP participation exists in all household surveys [37–39], so it is likely that some benefit recipients did not report in the ATUS interview that they were program participants. If this is the case, then our findings are an underestimate of the effects of the benefit cycle.

It is also possible that respondents underreported their eating occurrences. We did exclude the respondents with diaries flagged as “bad diaries,” which are likely to lack detail. In addition, starting in 2004, BLS included a prompt for eating if the respondent did not report any primary eating/drinking in his/her diary, minimizing the possibility of a respondent reporting no eating if they did engaged in eating/drinking on their diary day. Whereas it is still possible that respondents underreported all of their eating occurrences, the ATUS prompt makes it less likely that they are reporting no eating occurrences if they did in fact eat/drink on their diary day.

A discussion of the reliability of the American Time Use Survey estimates including sampling and nonsampling error is in the Technical Note in each American Time Use Survey News Release (http://www.bls.gov/tus/#news). A discussion of Eating & Health Module data and possible respondent under/overreporting is in the User’s Guide [27].

The ATUS and EHM data have information on primary eating/drinking occurrences and secondary eating occurrences, and the duration of those occurrences. These data do not have information on food intake, so we cannot make any conclusions about nutrition or calories consumed.

## Discussion

The benefit-cycle and the end-of-the-month problem are a concern in that SNAP participants may run out of funds and not have food after they redeem their benefits. We used time use data to investigate the most severe situation, no eating over the entire day, to see if SNAP participants had a greater likelihood of not eating over the day at the end of their benefit cycle.

We found that SNAP participants were increasingly more likely to experience a day with no eating occurrences after benefit issuance than nonparticipants. This is consistent with other researchers’ findings of an “end-of-the-month” problem, indicating that there is a monthly cycle in food consumption associated with the monthly benefit issuance policy. These results using time use data are consistent with previous research using food intake and consumer expenditure data [3,4]. Our contribution is using time diary data to determine probability of a day of no eating.

Because we analyzed the relatively rare but real occurrence of no eating—the most severe case of reduced food consumption—SNAP participants are more likely to incur hunger, and so may also suffer more health issues than other low-income individuals as a result of the benefit cycle.

Over 2009–13, the American Recovery and Reinvestment Act of 2009 increased SNAP monthly allotments. Previous research [8] found that this increase made SNAP benefits last longer over the month. However, now that those increases have expired, there is again concern about the benefit cycle.

One policy option that has been suggested to help smooth consumption over the benefit month is semi-monthly issuance of benefits. Now that all SNAP benefits are issued electronically, the option of semi-monthly benefits would be straightforward to implement. The policy could be an option that participants elect. A 1996 survey of food stamp participants found that respondents in households experiencing hunger were more likely than other participants to favor semi-monthly issuance [40].

The option of semi-monthly issuance would improve food choice architecture. Research in choice architecture [41,42]—influencing the choices people make by changing how the options are presented—has found that changing default options can change intertemporal behavior. This suggests that although the total amount of the benefits would be the same, splitting it into two payments would help overcome the tendency of individuals to be myopic and prefer the present to the future.

Allowing for the option of semi-monthly benefit issuance, along with the staggered issuance days that many states now have, may help smooth both participants’ consumption and retailers’ sales over the month.

## Supporting Information

S1 Appendix SNAP Issuances Dates Used (Actual and Imputed). (DOCX)

S2 Appendix Mean values of variables used in the simulation. (DOCX)

S3 Appendix Robustness check—Logit model of the probability of not eating over an average day, 2006–08, only states with issuance days in the first two weeks of the month. (DOCX)

S4 Appendix Robustness check—Logit model of the probability of not eating over an average day. 2006–08, estimated with rare event approach (Firth method) used.
(DOCX)
