# Supplementary material for: SNAP Participants’ Eating Patterns over the Benefit Month: A Time Use Perspective
Source: PLoS One. 2016 Jul 13;11(7):e0158422. doi: 10.1371/journal.pone.0158422 (PMC4943850; doi:10.1371/journal.pone.0158422)
Supplement: S1 Appendix — (DOCX) [file pone.0158422.s001.docx]

**S1 Appendix. SNAP Issuances Dates Used (Actual and Imputed)**

| **State** | **FIPS code** | **Actual issuance day(s) of month** | **Issuance day of month used** |
| --- | --- | --- | --- |
| AK | 2 | 1 | 1 |
| AL | 1 | 4-18 | 11 |
| AR | 5 | 4,5,8,9,10,11,12,13 | 8 |
| AZ | 4 | 1-13 | 7 |
| CA | 6 | 1-10 | 5 |
| CO | 8 | 1-10 | 5 |
| CT | 9 | 1-3 | 2 |
| DC | 11 | 1-10 | 5 |
| DE | 10 | 5-11 | 8 |
| FL | 12 | 1-15 | 8 |
| GA | 13 | 5-14 | 10 |
| HI | 15 | 1,3,5 but uncertainty in historical record | 4 |
| IA | 19 | 1-10 | 5 |
| ID | 16 | 1-5 | 3 |
| IL | 17 | 1,3,8,11,14,17,19,21,23 but uncertainty in historical record | 12 |
| IN | 18 | 1-10 | 5 |
| KS | 20 | 1-10 | 5 |
| KY | 21 | 1-10 | 5 |
| LA | 22 | 5-14 | 10 |
| MA | 25 | 1-14 | 7 |
| MD | 24 | 6-15 | 10 |
| ME | 23 | 10-14 | 12 |
| MI | 26 | 1-9 | 5 |
| MN | 27 | 4-13 | 8 |
| MO | 29 | 1-22 | 11 |
| MS | 28 | 5-19 | 12 |
| MT | 30 | 2-6 | 4 |
| NC | 37 | 3-12 | 7 |
| ND | 38 | 1 | 1 |
| NE | 31 | 1-5 | 3 |
| NH | 33 | 5 | 5 |
| NJ | 34 | 1-5 | 3 |
| NM | 35 | 1-20 | 10 |
| NV | 32 | 1 | 1 |
| NY* | 36 | 1-9 | 7 |
| OH | 39 | 1-10 | 5 |
| OK | 40 | 1 | 1 |
| OR | 41 | 1-9 | 5 |
| PA | 42 | 1-17, but depends on month | 9 |
| RI | 44 | 1 | 1 |
| SC | 45 | 1-10 | 5 |
| SD | 46 | 10 | 10 |
| TN | 47 | 1-10 | 5 |
| TX | 48 | 1-15 | 8 |
| UT | 49 | 5,11,15 | 11 |
| VA | 51 | 1 | 1 |
| VT | 50 | 1 | 1 |
| WA | 53 | 1-10 | 5 |
| WI | 55 | 2,3,5,6,8,9,11,12,14,15 | 8 |
| WV | 54 | 1-9 | 5 |
| WY | 56 | 1-4 | 2 |

* Weighted average of NY upstate (actual issuance days1-9) and NY City (days 1-15, but uncertainty in historical record).

Note: Actual issuance days are for 2006-08. Current issuance days are available at:

<http://www.fns.usda.gov/snap/snap-monthly-benefit-issuance-schedule>

Source: USDA Food and Nutrition Service.
