# Supplementary material for: SNAP Participants’ Eating Patterns over the Benefit Month: A Time Use Perspective
Source: PLoS One. 2016 Jul 13;11(7):e0158422. doi: 10.1371/journal.pone.0158422 (PMC4943850; doi:10.1371/journal.pone.0158422)
Supplement: S2 Appendix — (DOCX) [file pone.0158422.s002.docx]

**S2 Appendix. Mean values of variables used in the simulation.**

|  | Total Population | SNAP/FSP participants | Low-income non-SNAP | High-income, non-SNAP |
| --- | --- | --- | --- | --- |
| N | 32,060 | 2,292 | 7,489 | 21,475 |
| SNAP/FSP participant | 0.0656 | 1.0000 | 0 | 0 |
| ln(days since issuance) | 2.3288 | 2.3468 | 2.3012 | 2.3356 |
| ln(days since issuance) times SNAP/FSP participant | 0.1539 | 2.3468 | 0 | 0 |
| Year 2006 | 0.3373 | 0.3096 | 0.3432 | 0.3386 |
| Year 2007 | 0.3327 | 0.3603 | 0.3480 | 0.3243 |
| Saturday | 0.1451 | 0.1433 | 0.1434 | 0.1458 |
| Sunday | 0.1436 | 0.1470 | 0.1448 | 0.1432 |
| Holiday | 0.0180 | 0.0190 | 0.0164 | 0.0179 |
| Spring | 0.2500 | 0.2620 | 0.2385 | 0.2533 |
| Summer | 0.2485 | 0.2475 | 0.2504 | 0.2489 |
| Fall | 0.2524 | 0.2476 | 0.2570 | 0.2501 |
| Family income category (1-16) | 10.8682 | 5.7333 | 8.0767 | 12.3633 |
| Number of adults in household | 2.1752 | 2.1361 | 2.1646 | 2.1716 |
| Number of children in  household | 0.8243 | 1.5401 | 0.9143 | 0.7046 |
| Spouse/partner in household | 0.5843 | 0.3764 | 0.5176 | 0.6402 |
| Own home | 0.7545 | 0.3700 | 0.6248 | 0.8351 |
| Female | 0.5135 | 0.6537 | 0.5401 | 0.4883 |
| Employed | 0.6667 | 0.3877 | 0.5534 | 0.7477 |
| Age | 43.5364 | 39.1480 | 45.5048 | 43.5808 |
| Teen (age 15-19 years) | 0.0871 | 0.0875 | 0.0592 | 0.0803 |
| Age 65 years or over | 0.1404 | 0.0943 | 0.2116 | 0.1169 |
| Retired | 0.1376 | 0.0832 | 0.1973 | 0.1193 |
| Disabled | 0.0451 | 0.2254 | 0.0742 | 0.0170 |
| Less than high school diploma | 0.2884 | 0.3584 | 0.3845 | 0.2512 |
| Some college | 0.2562 | 0.1897 | 0.2331 | 0.2764 |
| College or advanced degree | 0.2700 | 0.0557 | 0.0962 | 0.3573 |
| African American | 0.1116 | 0.2950 | 0.1535 | 0.0774 |
| Asian | 0.0305 | 0.0170 | 0.0270 | 0.0333 |
| Hispanic | 0.1390 | 0.2368 | 0.2500 | 0.0874 |
| Metropolitan residence | 0.8192 | 0.7789 | 0.7890 | 0.8336 |
| West | 0.2282 | 0.1867 | 0.2307 | 0.2306 |
| South | 0.3580 | 0.4352 | 0.3938 | 0.3372 |
| Northeast | 0.1739 | 0.1498 | 0.1510 | 0.1841 |

Source: Authors’ estimates using the 2006-08 American Time Use Survey and Eating & Health Module data.
