# Supplementary material for: SNAP Participants’ Eating Patterns over the Benefit Month: A Time Use Perspective
Source: PLoS One. 2016 Jul 13;11(7):e0158422. doi: 10.1371/journal.pone.0158422 (PMC4943850; doi:10.1371/journal.pone.0158422)
Supplement: S3 Appendix — (DOCX) [file pone.0158422.s003.docx]

**S3 Appendix. Robustness check—Logit model of the probability of not eating over an average day, 2006-08, only states with actual issuance days in first two weeks of the month.**

|  | Maximum Likelihood  Estimate | Standard Error | Wald Chi Sq | Probability Chi Sq | Odds Ratio Estimate | 90% Wald CI min | 90% Wald CI max |
| --- | --- | --- | --- | --- | --- | --- | --- |
| Intercept | -3.4601 | 0.8224 | 17.7039 | <.0001 |  |  |  |
| SNAP characteristics |  |  |  |  |  |  |  |
| SNAP/FSP participant | -1.0396 | 0.6733 | 2.3842 | 0.1226 | 0.354 | 0.117 | 1.070 |
| ln(days since issuance) | -0.2326 | 0.1253 | 3.4449 | 0.0634 | 0.792 | 0.645 | 0.974 |
| ln(days since issuance) times SNAP/FSP participant | 0.5153 | 0.2567 | 4.0298 | 0.0447 | 1.674 | 1.098 | 2.554 |
| Calendar variables |  |  |  |  |  |  |  |
| Year 2006 | -0.2622 | 0.2692 | 0.9487 | 0.3301 | 0.769 | 0.494 | 1.198 |
| Year 2007 | -0.1836 | 0.2330 | 0.6209 | 0.4307 | 0.832 | 0.567 | 1.221 |
| Saturday | 0.5341 | 0.2322 | 5.2895 | 0.0215 | 1.706 | 1.164 | 2.499 |
| Sunday | 0.2429 | 0.2625 | 0.8565 | 0.3547 | 1.275 | 0.828 | 1.963 |
| Holiday | 0.8732 | 1.3436 | 0.4223 | 0.5158 | 2.395 | 0.263 | 21.830 |
| Spring | 0.1137 | 0.2555 | 0.1982 | 0.6562 | 1.120 | 0.736 | 1.706 |
| Summer | 0.3351 | 0.2732 | 1.5044 | 0.2200 | 1.398 | 0.892 | 2.191 |
| Fall | 0.4612 | 0.2972 | 2.4093 | 0.1206 | 1.586 | 0.973 | 2.586 |
| Household characteristics |  |  |  |  |  |  |  |
| Family income category (1-16) | -0.0638 | 0.0299 | 4.5497 | 0.0329 | 0.938 | 0.893 | 0.986 |
| Number of adults in household | -0.1305 | 0.1122 | 1.3514 | 0.2450 | 0.878 | 0.730 | 1.056 |
| Number of children in household | -0.0495 | 0.1100 | 0.2021 | 0.6530 | 0.952 | 0.794 | 1.141 |
| Spouse/partner in household | -0.3195 | 0.2594 | 1.5173 | 0.2180 | 0.726 | 0.474 | 1.113 |
| Own home | 0.0641 | 0.2546 | 0.0633 | 0.8014 | 1.066 | 0.701 | 1.621 |
| Individual characteristics |  |  |  |  |  |  |  |
| Female | -0.1045 | 0.1970 | 0.2812 | 0.5959 | 0.901 | 0.651 | 1.246 |
| Employed | 0.0848 | 0.2673 | 0.1006 | 0.7511 | 1.089 | 0.701 | 1.690 |
| Age | -0.0012 | 0.0117 | 0.0109 | 0.9170 | 0.999 | 0.980 | 1.018 |
| Teen (age 15-19 years) | -0.8034 | 0.6265 | 1.6441 | 0.1998 | 0.448 | 0.160 | 1.255 |
| Age 65 years or over | -0.3859 | 0.3984 | 0.9379 | 0.3328 | 0.680 | 0.353 | 1.309 |
| Retired | 0.0202 | 0.4617 | 0.0019 | 0.9651 | 1.020 | 0.477 | 2.181 |
| Disabled | -0.0302 | 0.4762 | 0.0040 | 0.9495 | 0.970 | 0.443 | 2.124 |
| High school diploma | 0.2840 | 0.3392 | 0.7010 | 0.4024 | 1.328 | 0.760 | 2.321 |
| Some college | -0.3134 | 0.3688 | 0.7220 | 0.3955 | 0.731 | 0.398 | 1.341 |
| College or advanced degree | -0.6168 | 0.4194 | 2.1632 | 0.1414 | 0.540 | 0.271 | 1.076 |
| African American | 0.7686 | 0.2409 | 10.1806 | 0.0014 | 2.157 | 1.451 | 3.205 |
| Asian | 0.7488 | 0.7126 | 1.1040 | 0.2934 | 2.114 | 0.655 | 6.828 |
| Hispanic | 0.5318 | 0.4172 | 1.6248 | 0.2024 | 1.702 | 0.857 | 3.380 |
| Region |  |  |  |  |  |  |  |
| Metropolitan residence | 0.0024 | 0.2296 | 0.0001 | 0.9916 | 1.002 | 0.687 | 1.462 |
| West | -0.3393 | 0.3616 | 0.8807 | 0.3480 | 0.712 | 0.393 | 1.291 |
| South | -0.3606 | 0.2626 | 1.8857 | 0.1697 | 0.697 | 0.453 | 1.074 |
| Northeast | -0.2964 | 0.2680 | 1.2226 | 0.2689 | 0.744 | 0.478 | 1.155 |
| N | 23,221 |  |  |  |  |  |  |
| Percent of observations that have no eating occurrences | 0.7% |  |  |  |  |  |  |
| Likelihood Ratio, Pr>ChiSq | <.0001 |  |  |  |  |  |  |
| Score, Pr>ChiSq | <.0001 |  |  |  |  |  |  |
| Wald, Pr>ChiSq | <.0001 |  |  |  |  |  |  |
| Association of predicted and observed: 64.3 percent Concordant, 25.9 Discordant, 9.8 Tied. | | | | | | | |

Note: Age 15 and over. 90% Wald CI min=the minimum value of the Wald confidence interval at the 90% level. 90% Wald CI max=the maximum value of the Wald confidence interval at the 90% level. Family income categories are: 1=Less than $5,000; 2=$5,000 to $7,499; 3=$7,500 to $9,999; 4=$10,000 to $12,499; 5=$12,500 to $14,999; 6=$15,000 to $19,999; 7=$20,000 to $24,999; 8=$25,000 to $29,999; 9=$30,000 to $34,999; 10=$35,000 to $39,999; 11=$40,000 to $49,999; 12=$50,000 to $59,999; 13=$60,000 to $74,999; 14=$75,000 to $99,999; 15=100,000 to $149,999; and 16=$150,000 and over. Reference group is SNAP/FSP non-participant, year 2008, non-holiday weekday, winter, no spouse/partner in home, do not own home, male, not employed, age 20-64 years, not retired, not disabled, less than high school diploma, white non-Hispanic, nonmetropolitan area, and Midwest. Concordant-Discordant is a measure of the model’s performance. For more information, see Paul D. Allison, Logistic Regression Using the SAS System: Theory and Application, Cary, NC: SAS Institute Inc., 1999.

States with actual issuances days in the first two weeks of the month: AK, AZ, AR, CA, CO, CT, DE, DC, GA, HI, ID, IN, IA, KS, KY, LA, ME, MA, MI, MN, MT, NE, NV, NH, NJ, NY, NC, ND, OH, OK, OR, PA, RI, SC, SD, TN, VT, VA, WA, WV, and WY.

Source: Authors’ estimates using 2006-08 American Time Use Survey and Eating & Health Module data.
