# Supplementary material for: SNAP Participants’ Eating Patterns over the Benefit Month: A Time Use Perspective
Source: PLoS One. 2016 Jul 13;11(7):e0158422. doi: 10.1371/journal.pone.0158422 (PMC4943850; doi:10.1371/journal.pone.0158422)
Supplement: S4 Appendix — 2006–08, estimated with rare event approach (Firth method) used. (DOCX) [file pone.0158422.s004.docx]

**S4 Appendix. Robustness check—Logit model of the probability of not eating over an average day, 2006-08, estimated with rare event approach (Firth method) used.**

|  | Maximum Likelihood  Estimate | Standard Error | Wald Chi Sq | Probability Chi Sq | Odds Ratio Estimate | 90% Wald CI min | 90% Wald CI max |
| --- | --- | --- | --- | --- | --- | --- | --- |
| Intercept | -3.5906 | 0.0039 | 856622.0 | <.0001 |  |  |  |
| SNAP characteristics |  |  |  |  |  |  |  |
| SNAP/FSP participant | -1.1027 | 0.0051 | 46691.6 | <.0001 | 0.332 | 0.329 | 0.335 |
| ln(days since issuance) | -0.1435 | 0.0005 | 84143.2 | <.0001 | 0.866 | 0.866 | 0.867 |
| ln(days since issuance) times SNAP/FSP participant | 0.4444 | 0.0019 | 57281.4 | <.0001 | 1.560 | 1.555 | 1.564 |
| Calendar variables |  |  |  |  |  |  |  |
| Year 2006 | -0.0185 | 0.0012 | 222.4 | <.0001 | 0.982 | 0.980 | 0.984 |
| Year 2007 | -0.1747 | 0.0013 | 18182.0 | <.0001 | 0.840 | 0.838 | 0.841 |
| Saturday | 0.4930 | 0.0013 | 140497.3 | <.0001 | 1.637 | 1.634 | 1.641 |
| Sunday | 0.2297 | 0.0015 | 24914.0 | <.0001 | 1.258 | 1.255 | 1.261 |
| Holiday | 0.6066 | 0.0031 | 38960.9 | <.0001 | 1.834 | 1.825 | 1.843 |
| Spring | -0.3262 | 0.0016 | 43737.6 | <.0001 | 0.722 | 0.720 | 0.724 |
| Summer | 0.0081 | 0.0014 | 31.8 | <.0001 | 1.008 | 1.006 | 1.011 |
| Fall | 0.0860 | 0.0014 | 3738.4 | <.0001 | 1.090 | 1.087 | 1.092 |
| Household characteristics |  |  |  |  |  |  |  |
| Family income category (1-16) | -0.0535 | 0.0002 | 97430.7 | <.0001 | 0.948 | 0.948 | 0.948 |
| Number of adults in household | -0.1038 | 0.0006 | 25221.5 | <.0001 | 0.901 | 0.900 | 0.902 |
| Number of children in household | -0.0110 | 0.0005 | 455.4 | <.0001 | 0.989 | 0.988 | 0.990 |
| Spouse/partner in household | -0.3717 | 0.0013 | 84014.8 | <.0001 | 0.690 | 0.688 | 0.691 |
| Own home | -0.0237 | 0.0013 | 324.3 | <.0001 | 0.977 | 0.974 | 0.979 |
| Individual characteristics |  |  |  |  |  |  |  |
| Female | -0.0951 | 0.0011 | 7934.6 | <.0001 | 0.909 | 0.908 | 0.911 |
| Employed | -0.0184 | 0.0015 | 150.3 | <.0001 | 0.982 | 0.979 | 0.984 |
| Age | 0.0042 | 0.0001 | 6415.1 | <.0001 | 1.004 | 1.004 | 1.004 |
| Teen (age 15-19 years) | -0.0347 | 0.0024 | 208.7 | <.0001 | 0.966 | 0.962 | 0.970 |
| Age 65 years or over | -0.3583 | 0.0028 | 16681.9 | <.0001 | 0.699 | 0.696 | 0.702 |
| Retired | -0.4637 | 0.0030 | 24708.8 | <.0001 | 0.629 | 0.626 | 0.632 |
| Disabled | -0.3039 | 0.0026 | 13905.7 | <.0001 | 0.738 | 0.735 | 0.741 |
| High school diploma | 0.0618 | 0.0015 | 1616.6 | <.0001 | 1.064 | 1.061 | 1.066 |
| Some college | -0.4021 | 0.0018 | 50804.4 | <.0001 | 0.669 | 0.667 | 0.671 |
| College or advanced degree | -0.8644 | 0.0022 | 159614.1 | <.0001 | 0.421 | 0.420 | 0.423 |
| African American | 0.8614 | 0.0013 | 411079.9 | <.0001 | 2.367 | 2.361 | 2.372 |
| Asian | 0.4614 | 0.0032 | 20107.8 | <.0001 | 1.586 | 1.578 | 1.595 |
| Hispanic | 0.2041 | 0.0016 | 15558.0 | <.0001 | 1.226 | 1.223 | 1.230 |
| Region |  |  |  |  |  |  |  |
| Metropolitan residence | -0.0004 | 0.0014 | 0.1 | 0.7888 | 1.000 | 0.997 | 1.002 |
| West | -0.2240 | 0.0016 | 18889.1 | <.0001 | 0.799 | 0.797 | 0.801 |
| South | -0.2218 | 0.0013 | 27217.8 | <.0001 | 0.801 | 0.799 | 0.803 |
| Northeast | -0.1803 | 0.0017 | 11727.6 | <.0001 | 0.835 | 0.833 | 0.837 |
| N | 32,060 |  |  |  |  |  |  |
| Percent of observations that have no eating occurrences | 0.7% |  |  |  |  |  |  |
| Likelihood Ratio, Pr>ChiSq | <.0001 |  |  |  |  |  |  |
| Score, Pr>ChiSq | <.0001 |  |  |  |  |  |  |
| Wald, Pr>ChiSq | <.0001 |  |  |  |  |  |  |
| Association of predicted and observed: 62.6 percent Concordant, 26.5 Discordant, 10.9 Tied. | | | | | | | |

Note: Age 15 and over. 90% Wald CI min=the minimum value of the Wald confidence interval at the 90% level. 90% Wald CI max=the maximum value of the Wald confidence interval at the 90% level. Family income categories are: 1=Less than $5,000; 2=$5,000 to $7,499; 3=$7,500 to $9,999; 4=$10,000 to $12,499; 5=$12,500 to $14,999; 6=$15,000 to $19,999; 7=$20,000 to $24,999; 8=$25,000 to $29,999; 9=$30,000 to $34,999; 10=$35,000 to $39,999; 11=$40,000 to $49,999; 12=$50,000 to $59,999; 13=$60,000 to $74,999; 14=$75,000 to $99,999; 15=100,000 to $149,999; and 16=$150,000 and over. Reference group is SNAP/FSP non-participant, year 2008, non-holiday weekday, winter, no spouse/partner in home, do not own home, male, not employed, age 20-64 years, not retired, not disabled, less than high school diploma, white non-Hispanic, nonmetropolitan area, and Midwest. Concordant-Discordant is a measure of the model’s performance. For more information, see Paul D. Allison, Logistic Regression Using the SAS System: Theory and Application, Cary, NC: SAS Institute Inc., 1999.

Source: Authors’ estimates using 2006-08 American Time Use Survey and Eating & Health Module data.
